# Differential Impact of Single-Dose Fe Ion and X-Ray Irradiation on Endothelial Cell Transcriptomic and Proteomic Responses

**DOI:** 10.3389/fphar.2017.00570

**Published:** 2017-09-22

**Authors:** Bjorn Baselet, Omid Azimzadeh, Nadine Erbeldinger, Mayur V. Bakshi, Till Dettmering, Ann Janssen, Svetlana Ktitareva, Donna J. Lowe, Arlette Michaux, Roel Quintens, Kenneth Raj, Marco Durante, Claudia Fournier, Mohammed A. Benotmane, Sarah Baatout, Pierre Sonveaux, Soile Tapio, An Aerts

**Affiliations:** ^1^Radiobiology Unit, Institute for Environment, Health and Safety, Belgian Nuclear Research Centre (SCK•CEN) Mol, Belgium; ^2^Pole of Pharmacology and Therapeutics, Institut de Recherche Expérimentale et Clinique, Université catholique de Louvain Brussels, Belgium; ^3^Institute of Radiation Biology, Helmholtz Zentrum Munich, German Research Center for Environmental Health Munich, Germany; ^4^GSI Helmholtz Centre for Heavy Ion Research Darmstadt, Germany; ^5^Technical University Darmstadt Darmstadt, Germany; ^6^Department of Radiation Effects, Centre for Radiation, Chemical and Environmental Hazards, Public Health England Didcot, United Kingdom; ^7^Department of Molecular Biotechnology, Ghent University Ghent, Belgium

**Keywords:** irradiation, radiotherapy, X-rays, Fe ions, linear energy transfer, endothelial cells, cardiovascular disease

## Abstract

**Background and Purpose:** Radiotherapy is an essential tool for cancer treatment. In order to spare normal tissues and to reduce the risk of normal tissue complications, particle therapy is a method of choice. Although a large part of healthy tissues can be spared due to improved depth dose characteristics, little is known about the biological and molecular mechanisms altered after particle irradiation in healthy tissues. Elucidation of these effects is also required in the context of long term space flights, as particle radiation is the main contributor to the radiation effects observed in space. Endothelial cells (EC), forming the inner layer of all vascular structures, are especially sensitive to irradiation and, if damaged, contribute to radiation-induced cardiovascular disease.

**Materials and Methods:** Transcriptomics, proteomics and cytokine analyses were used to compare the response of ECs irradiated or not with a single 2 Gy dose of X-rays or Fe ions measured one and 7 days post-irradiation. To support the observed inflammatory effects, monocyte adhesion on ECs was also assessed.

**Results:** Experimental data indicate time- and radiation quality-dependent changes of the EC response to irradiation. The irradiation impact was more pronounced and longer lasting for Fe ions than for X-rays. Both radiation qualities decreased the expression of genes involved in cell-cell adhesion and enhanced the expression of proteins involved in caveolar mediated endocytosis signaling. Endothelial inflammation and adhesiveness were increased with X-rays, but decreased after Fe ion exposure.

**Conclusions:** Fe ions induce pro-atherosclerotic processes in ECs that are different in nature and kinetics than those induced by X-rays, highlighting radiation quality-dependent differences which can be linked to the induction and progression of cardiovascular diseases (CVD). Our findings give a better understanding of the underlying processes triggered by particle irradiation in ECs, a crucial aspect for the development of protective measures for cancer patients undergoing particle therapy and for astronauts in space.

## Introduction

The main goal of radiotherapy is to efficiently target and eradicate tumors while sparing surrounding healthy tissues (Bentzen, 2006; Barnett et al., 2009). One of the recent developments in radiotherapy modalities is high linear energy transfer (LET) particle therapy (Stone et al., 2003). In this technique, accelerated charged particle beams, such as proton and carbon ions, are used (Wilson, 1946), which have improved depth dose characteristics in comparison to low LET radiotherapy (Durante and Loeffler, 2010). In this way, tumors can be irradiated with great precision, minimizing the dose to surrounding healthy tissues. Besides their physical advantage, charged particle beams induce more damage per unit of dose, i.e., demonstrate a higher relative biological effectiveness (RBE) compared to conventional low LET X-ray radiotherapy (Kramer et al., 2003; Schulz-Ertner and Tsujii, 2007). Despite this advantage, particle therapy is not commonly used due to its complex and expensive nature. Consequently, particle therapy currently focuses on localized tumors in proximity to critical organs and tumors resistant to conventional treatments, such as uveal melanoma, pediatric tumors, head-and-neck cancer and prostate cancer (Schulz-Ertner and Tsujii, 2007; Durante and Loeffler, 2010). In the last 10 years, however, there has been an exponential growth of new facilities (Jermann, 2015), a rapid increase in the number of patients treated (Jermann, 2015) and several new indications including lung cancer (Chang et al., 2016), breast cancer (Akamatsu et al., 2014), and pancreatic cancer (Nichols et al., 2015). Nonetheless, due to the limited number of patients treated with particle therapy at present, induction of normal tissue damage cannot be predicted correctly as molecular pathways and biological functions altered by high LET radiotherapy are still largely unknown (Schulz-Ertner and Tsujii, 2007; Durante and Loeffler, 2010; Newhauser and Durante, 2011). Identification and characterization of these processes is not only imperative for the development of protective measures for cancer patients undergoing high LET radiotherapy, but also for astronauts in space. Indeed, high LET particles are omnipresent in space as part of cosmic radiation. Among these particles, iron (Fe) ions are the major contributor to biological radiation effects due to their high LET value (Moreels et al., 2012; Fernandez-Gonzalo et al., 2017).

One of the known consequences of conventional low LET radiotherapy is an increased risk of cardiovascular diseases (CVD), especially when the heart is located within the radiation field (Darby et al., 2013; Aleman et al., 2014). For example, low LET radiation exposure during breast cancer therapy has been shown to accelerate atherosclerosis leading to CVD (Darby et al., 2013). CVD is an umbrella term for several types of disorders that affect the heart, such as cardiomyopathy and heart failure, or blood vessels, such as coronary artery disease (Kumar et al., 2013). Although many aspects of the mechanisms by which ionizing radiation (IR) causes CVD are still unknown, evidence indicates that it acts at least in part by inducing endothelial cell (EC) dysfunction leading to increased oxidative stress, inflammation, coagulation, senescence and EC death (Vita and Keaney, 2002; Corre et al., 2013; Rombouts et al., 2013, 2014; Yentrapalli et al., 2013a,b; Widmer and Lerman, 2014; Azimzadeh et al., 2015; Baselet et al., 2017). These responses may eventually lead to the onset and/or progression of atherosclerosis, the leading cause of CVD (Schultz-Hector and Trott, 2007; Borghini et al., 2013). Compared to low LET, even less is known about the effects of high LET irradiation in the context of radiation-induced CVD. High LET irradiation was nevertheless found to be more damaging for ECs (Grabham et al., 2011; Helm et al., 2016) and to inhibit blood vessel formation differently than low LET irradiation (Grabham et al., 2013). Furthermore, atherogenesis was accelerated in obese apoE^−/−^ mice at both 13 and 40 weeks after exposure to 2 and 5 Gy Fe ions (Yu et al., 2011). In rats, 1 Gy of Fe ions has been shown to relaxation, increased reactive oxygen species levels and decreased nitric oxide production, indicating endothelial dysfunction, which was associated with increased aortic stiffness and impaired endothelial-dependent (Soucy et al., 2011). Taking into account the increasing use of high LET radiation in cancer therapy (Jermann, 2015), there is an obvious necessity to further study its possible cardiovascular effects.

In this study, we hypothesized that high LET exposure would induce more EC dysfunction related processes in exposed ECs when compared to low LET exposure. Therefore, we aimed to compare the transcriptomic, proteomic and pro-inflammatory response of ECs irradiated with either Fe ions or X-rays. We identified differences in signaling pathways and response mechanisms influenced by low (X-ray) compared to high (Fe ion) LET radiation in telomerase-immortalized human coronary artery ECs. ECs were irradiated or not with a single dose of 2 Gy and changes were measured after 1 and 7 days. We conducted both transcriptomic and proteomic analyses, which were complemented with pro-inflammatory cytokine quantification and monocyte adhesion tests on irradiated ECs. We report time- and radiation quality-dependent changes in the EC response after exposure to IR.

## Materials and methods

### Cells, reagents and irradiation

ECs from human coronary artery were purchased from European Cell Culture Collection (HCAECs Cat. No: 300-05) and transduced with retroviruses bearing the est2 gene, a yeast homolog of the human TERT protein. ECs were grown in Human MesoEndo Endothelial Cell Medium (Cat. No. 212-500, Cell Applications) and cultured at 37°C with 5% CO_2_ in a humidified incubator as described previously (Lowe and Raj, 2014). Cell were counted (Beckman Coulter counter) and seeding was carried out 2 days prior to irradiation with 40,000 cells/cm^2^ in 0.4 ml medium/cm^2^. This resulted in a confluence of 90–100% at the time of irradiation visualized on a Leica DMI4000b microscope (Leica Microsystems). ECs were exposed to 2 Gy of X-ray irradiation using an AGO HS320/250 X-ray cabinet (250 kV, 13 mA, 1.5 mm Al, 1.2 mm Cu, 3 KeV/μm) or Fe ions (1 GeV/u, 155 keV/μm), both with a dose rate of 1.5 Gy/min. Fe ion irradiation of EC monolayers was performed in the entrance channel of the beam to mimic the effects on healthy tissues. 1 GeV Fe ions occurs in the plateau region of the depth dose profile and has a penetration depth of 25 cm in water (Scholz, 2003; Lee et al., 2011). Cells were not passaged during experiments to simulate normal, quiescent endothelium, but medium was changed regularly to ensure viability (three times per week after X-ray and twice per week after Fe ion irradiation).

### Transcriptomic analysis

#### Microarray preparation, analysis, and statistics

Total RNA of ECs was extracted according to manufacturer's instructions using the AllPrep DNA/RNA/protein mini kit (Qiagen). RNA was quantified using a NanoDrop Spectrophotometer and its quality assessed with an Agilent 2100 Bioanalyzer. Samples with a RNA integrity number > 8 were used for hybridization onto Affymetrix Human Gene 2.0 ST arrays, following manufacturer's instructions. Raw data were uploaded to the Partek Genomics Suite (version 6.6) and normalized using a customized Robust Multi-chip Analysis algorithm (background correction for entire probe sequence, quantile normalization, log2 transformation of intensity signals). Microarray data were filtered to exclude genes expressed below the background signal in at least 67% of all samples and analyzed using three-way ANOVA. Differentially expressed genes were identified as those with a fold change > |1.2| and *p* < 0.05 after correction for multiple testing according to Benjamini and Hochberg (1995). The data have been deposited in the ArrayExpress database (http://www.ebi.ac.uk/arrayexpress; accession number E-MTAB-5754).

#### Functional enrichment analysis

Functional gene enrichment was performed and visualized using the GOrilla tool (Eden et al., 2007, 2009). Settings were: organism: *Homo sapiens*; running mode: two unranked lists of genes (target list: differentially expressed genes, background list: genes expressed above background in at least 33% of all samples); *p* < 0.001. To exclude redundant gene ontology terms, results were reduced using REViGO (Rudjer Boskovic Institute, Croatia) with an allowed similarity of 0.4 (Supek et al., 2011). The version of Gene Ontology used was go_201507-termdb.obo-xml.gz (http://archive.geneontology.org/full/2015-07-01/).

### Proteomic analysis

#### Protein labeling

Protein labeling with isotope-coded protein labels (ICPL) was done as previously reported (Azimzadeh et al., 2013). Briefly, triplicate aliquots of 25 μg of cell lysate proteins obtained from either sham or irradiated ECs were labeled with ICPL reagents (SERVA) following manufacturer's instructions. ICPL0 was used for sham-irradiated ECs and ICPL6 for irradiated cells. Labeling was done using three biological replicates per condition. Heavy and light labeled samples were combined and separated by 12% SDS gel electrophoresis before staining with colloidal Coomassie solution.

#### Protein analysis

After staining, SDS-PAGE lanes were cut into 5 slices and subjected to in-gel digestion with trypsin (SERVA), as previously described (Merl et al., 2012). Digested peptides were separated by reversed phase liquid chromatography (LC), and mass spectrometry (MS) analyses were done with a linear quadrupole ion trap-Orbitrap (LTQ Orbitrap XL) mass spectrometer (ThermoFisher) equipped with a nano-ESI source (Hauck et al., 2010). This method allowed for sequential isolation of up to 10 most intense ions, depending on signal intensity, for fragmentation on the linear ion trap using collision-induced dissociation at a target value of 100,000 ions. High resolution MS scans in the Orbitrap and MS/MS scans in the linear ion trap were performed in parallel. Target peptides already selected for MS/MS were dynamically excluded for 30 s. Acquired MS/MS spectra were searched against the Ensembl Human database using Mascot (Matrix Science, version 2.3.02; 20140909, Number of sequences: 100607) with the following parameters: MS/MS spectra were searched with a precursor mass tolerance of 10 ppm and a fragment tolerance of 0.8 Da; Arg-C was selected as enzyme. One missed cleavage was allowed, and carbamidomethylation was set as a fixed modification. Oxidized methionine and the heavy and light ICPL labeled lysines as well as heavy and light labels of proteins were set as variable modifications.

#### Protein identification and quantification

Data processing for protein identification and quantification of ICPL pairs was performed using Proteome Discoverer version 1.3 (Thermo Fisher). This software provides automated strict statistical analysis of the protein quantification using unique peptides only. To minimize experimental bias, the software was set to normalize on the protein median (minimum protein count: 20). Complete peptide and protein profiles were filtered using high peptide confidence and top one peptide rank filters. False discovery rate (FDR) was calculated at the peptide level for all experimental runs using the Decoy option in Mascot; this rate was estimated to be lower than 1% using the identity threshold as the scoring threshold system. Differentially labeled isotopic pairs were detected with a mass precision of 2 ppm and a retention time window of 0.3 min. Proteins identified by at least two unique peptides in two out of three replicates, with ratios of heavy / light (H/L) label > 1.50-fold or < 0.66-fold (*t*-test, *p* < 0.05) were defined as significantly differentially expressed. Raw MS data have been deposited in the STORE^DB^ database (http://www.storedb.org; dataset identifier doi: 10.20348/STOREDB/1086).

#### Protein-protein interaction and signaling networks

Analysis of possible protein-protein interactions and signaling networks was performed with the INGENUITY Pathway Analysis (IPA) software tool (INGENUITY System, http://www.INGENUITY.com). IPA is a knowledge database generated from peer-reviewed scientific publications that enables to discover highly represented functions and pathways (*p* < 0.001) from large quantitative data sets (Wu et al., 2007).

#### Western blotting

For the validation of proteomics data, 10 μg of cell protein extract was separated on 12% SDS polyacrylamide gels according to Laemmli (1970) before being transferred onto PVDF membranes and labeled with primary antibodies against caveolin-1 (#3238, cell signaling) and α-tubulin (#GTX72291, GeneTex). Secondary antibodies were horseradish peroxidase-conjugated anti-rabbit and anti-mouse, respectively, and was detected usingthe ECL system (GE Healthcare). Signals were quantified using ImageJ software and normalized to the α-tubulin expression.

### Functional analyses

#### Cytokine release assays

The release of interleukin (IL)-6, IL-8 and C-C motif chemokine ligand 2 (CCL2) was determined with enzyme-linked immunosorbent assays (ELISAs; eBioscience). Cell culture supernatant was collected at the indicated time points after irradiation and frozen at −80°C. Medium was replaced 24 h before each time point to ensure cell viability and consistency. ELISAs were performed according to manufacturer's instructions. Measured cytokine concentrations were normalized to the cell number (Beckman Coulter counter), as total protein content per cell was dependent on irradiation dose, and the volume of supernatant at the time of collection, and calculated as fold change compared to control cells at matched time points.

#### Monocyte adhesion assay

Monocyte adhesion on ECs was tested as previously described (Lowe and Raj, 2014, 2015). In brief, ECs were seeded on fibronectin-coated glass coverslips and cultured until confluent. Seven days after irradiation, 500,000 Cell Tracker-labeled HL-60 monocytes (ThermoFisher Scientific) were added to the coverslip and incubated at 37°C. After 30 min, coverslips were washed with HBSS, fixed in formalin and embedded in Vectashield Hard Set mounting medium with 4′6-diamidino-2-phenylindole (Vectorlabs H-1500). Monocytes adhering to ECs were microscopically quantified.

#### Statistics

The data show means ± SEM. Differences between sham and irradiated samples at different time-points were determined by two-sided *t*-test (Welch-test). X-ray and Fe ion data are shown from three to one independent experiments, respectively. For Fe ion experiments, data represent 2 biological replicates with 2–3 technical replicates each. For X-ray experiments, data represent 9 biological replicates with 3 technical replicates each. *P* < 0.05 was considered to be statistically significant.

## Results

### The transcriptional response of Fe ion-irradiated ECs is more pronounced and persistent than the X-ray radiation response

**Figure 1:**
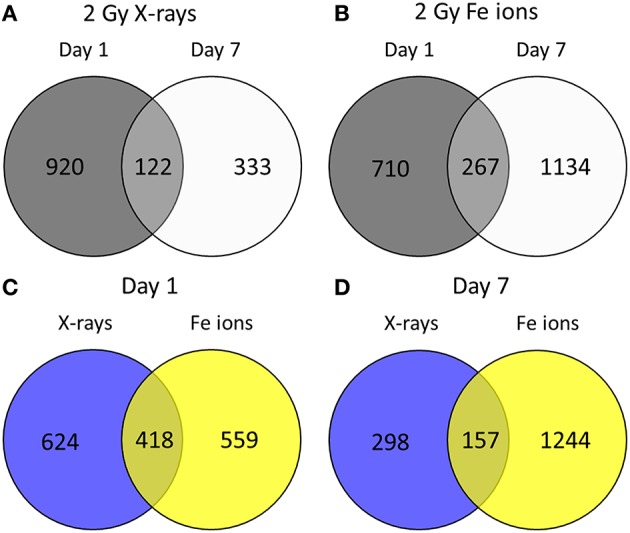
Fe ion irradiation induces a more pronounced and persistent transcriptional response in ECs in comparison to X-ray irradiation. Venn diagrams indicate the number of differentially expressed genes after exposure to a single 2.0 Gy dose of X-rays **(A)** or Fe ions **(B)**. The gene expression changes after low and high LET irradiation are compared on day 1 **(C)** and on day 7 **(D)**. Changes are compared gene expression in sham irradiated cells, as described in section Materials and Methods (*N* = 3, *n* = 12).

Little is known about the effects of high LET irradiation in the context of radiation-induced CVD. To gain more knowledge on the molecular pathways affected high LET irradiation, we tested whether irradiation of ECs with a single 2 Gy Fe ion dose induced different effects after 1 and 7 days in comparison to a single 2 Gy X-ray dose. Using a genome-wide gene expression analysis, we observed time- and radiation quality-dependent changes in gene expression. Differentially expressed genes (up or down-regulated) using Fe ion or X-ray irradiation are listed in Supplementary Data File 1. The number of deregulated genes and the shared deregulated genes between radiation qualities or time points are shown in Figure 1. A single X-ray dose of 2 Gy caused a profound effect on the gene expression on day 1 (1042 genes), which significantly decreased on day 7 (455 genes) (Figure 1A). Comparatively, Fe ions caused a smaller effect on gene expression at day 1 (977 genes), but this effect increased in size at day 7 (1401 genes) and demonstrated a larger overlap between both time points (Figure 1B). When comparing the samples irradiated with either X-rays or Fe ions, a lot of differentially expressed genes were shared at day 1 (418 genes), which was lost for the most part at day 7 (157 genes) (Figures 1C,D).

### Irradiation of ECs represses the expression of genes involved in cell cycling and cell-cell adhesion

**Figure 2:**
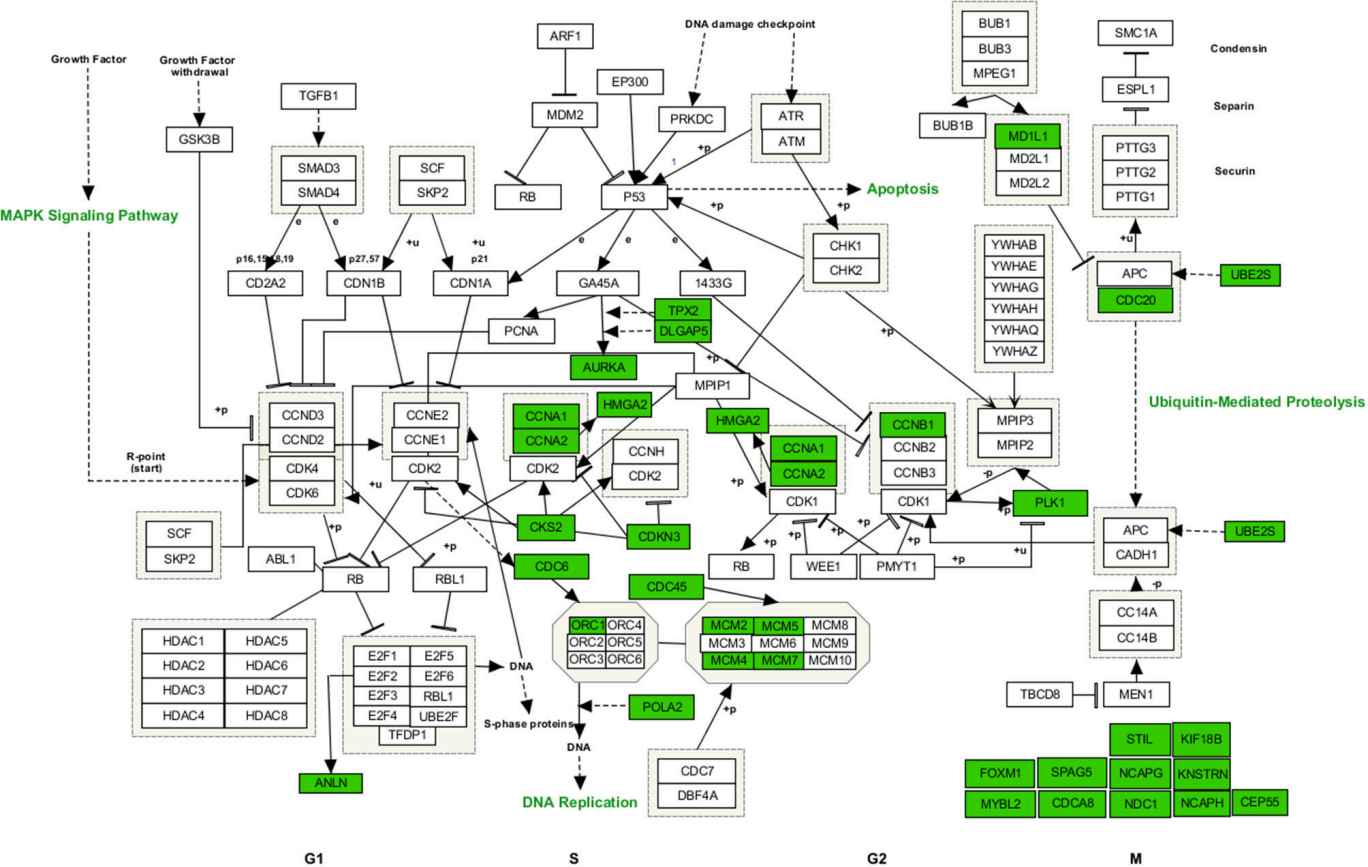
Upregulated genes involved in cell cycle regulated processes at 7 days after a single dose of 2 Gy X-rays. Arborescence diagram indicates cell cycle pathway with the identified upregulated genes indicated in green. Pathway diagram was adapted from Wikipathways and modified with Pathvisio. Changes are shown compared to gene expression in sham irradiated cells (*N* = 3, *n* = 12).

**Figure 3:**
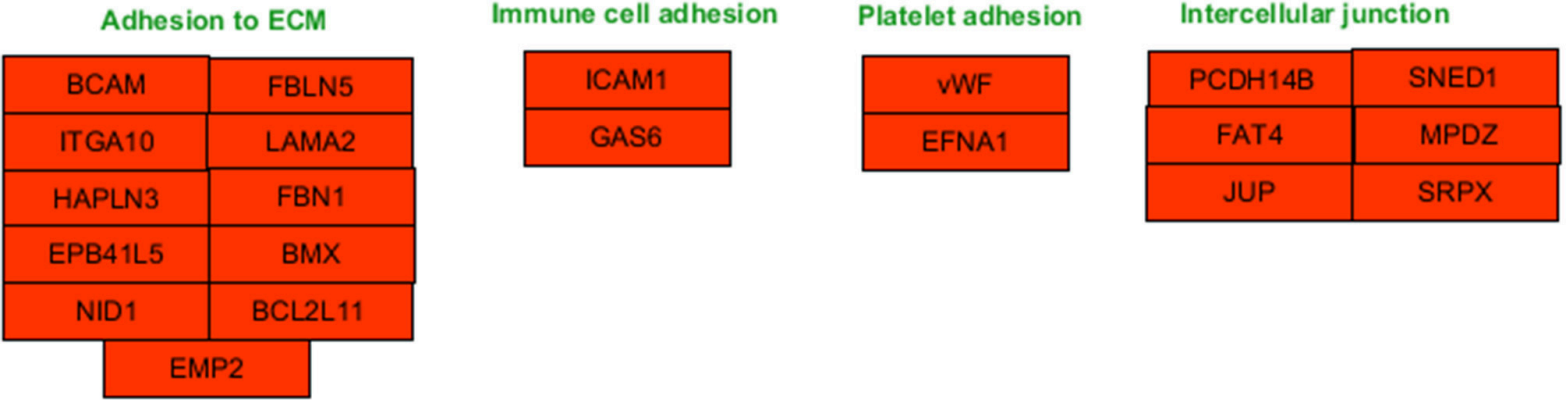
Downregulated genes involved in cell-cell adhesion processes at 1 day after a single dose of 2 Gy X-rays. Arborescence diagram indicates cell-cell adhesion pathway with the identified downregulated genes indicated in red. Pathway diagram was adapted from Wikipathways and modified with Pathvisio. Changes are shown compared to gene expression in sham irradiated cells (*N* = 3, *n* = 12).

**Figure 4:**
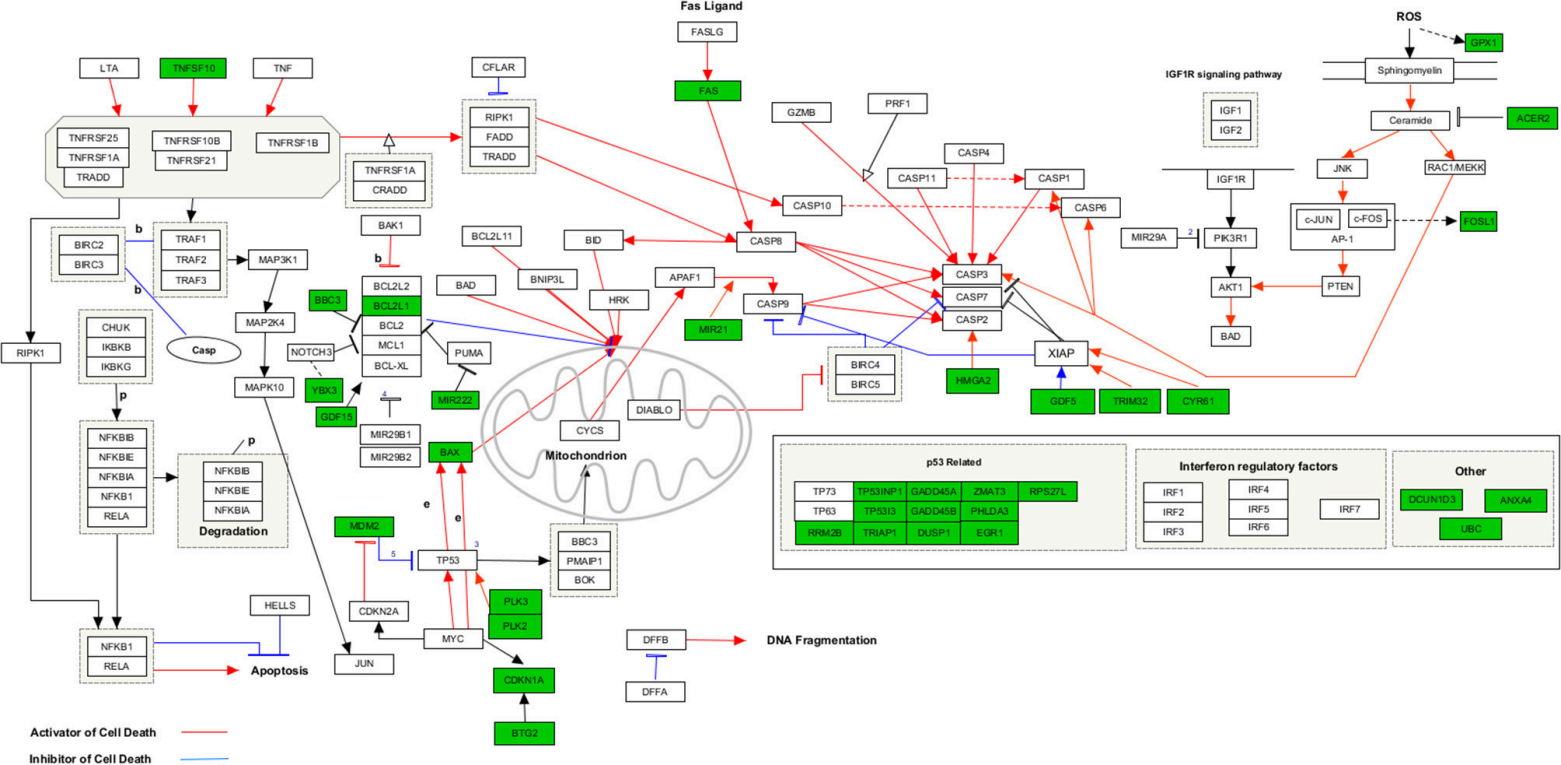
Upregulated genes involved in apoptosis signaling at 1 day after a single dose of 2 Gy Fe ions. Arborescence diagram indicates apoptosis pathway with the identified upregulated genes indicated in green. Pathway diagram was adapted from Wikipathways and modified with Pathvisio. Changes are shown compared to gene expression in sham irradiated cells (*N* = 3, *n* = 12).

Functional enrichment analysis revealed that, 1 day after exposure to a single X-ray dose of 2 Gy, upregulated differentially expressed genes were involved in cell-cell adhesion, whereas downregulated differentially expressed genes in cell cycle-related processes, such as DNA replication and chromosome segregation (Table 1, upper panel and Supplementary Data File 2). On day 7 after exposure to a single X-ray dose of 2 Gy, gene ontology analysis highlighted the acute nature of the induced transcriptional changes. Upregulated differentially expressed genes were involved in cell cycle-related processes (top 10 upregulated: *CCNA1, UBE2S, CDC6, MAD2L1, QRC1, AURKA, MCM4, CKS2, SPAG5*, and *CDCA8*; Figure 2), whereas downregulated differentially expressed genes played a role in cell-cell adhesion (top 10 downregulated: *VWF, SNED1, ITGA10, ICAM1, FAT4, LAMA2, PCDHB14, HAPLN3, FBLN5*, and *BCAM;* Figure 3) (Table 1, lower panel). The transcriptional response to low LET irradiation was then compared to that of a same 2 Gy dose of high LET IR. On day 1, Fe ion-exposed ECs upregulated the expression of genes involved in cell cycle arrest, such as genes related to p53 DNA damage signal transduction, which was associated to a downregulation of genes involved in cell cycle-related processes, such as DNA replication and chromosome segregation (Table 2, upper panel). Fe ions also upregulated the expression of genes controlling apoptosis at day 1 post-irradiation (top 10 upregulated: *MIR21, YBX3, MDM2, MIR222, ACER2, TP53INP1, ZMAT3, BTG2, GDF5*, and *PHLDA3;* Figure 4). Seven days after exposure to a single 2 Gy dose of Fe ions, ECs demonstrated a downregulation of gene expression involved in cell cycle-related processes, indicating a persistent cell cycle response (Table 2, lower panel). As opposed to day 1, downregulation of the expression of genes involved in cell death regulation and cell-cell adhesion was observed.

**Table 1:** X-ray irradiation of ECs induces an acute gene expression signature suggestive of cycle block and increased cellular adhesion.^*^

	Upregulated description of process	−log10 (*p*-value)	Downregulated description of process	−log10 (*p*-value)
2 Gy X-rays Day 1	Regulation of multicellular organismal process	9	Cell cycle process	111
	Regulation of localization	9	Chromosome organization	76
	Signal transduction	9	DNA metabolic process	54
	Regulation of cellular component movement	8	Cell division	50
	Regulation of locomotion	8	DNA replication	44
	Cell adhesion	7	Cellular component organization or biogenesis	36
	Biological adhesion	7	Chromosome segregation	26
	Response to stimulus	7	Microtubule-based process	19
	Regulation of cell proliferation	7	Regulation of chromosome segregation	18
	Developmental process	7	DNA synthesis involved in DNA repair	18
2 Gy X-rays Day 7	Mitotic cell cycle process	14	Cell-substrate adhesion	7
	Cell division	10	Extracellular matrix organization	4
	Chromosome organization	9	Extracellular structure organization	4
	DNA replication initiation	9	Ethanol oxidation	4
	Cell proliferation	6	Biological adhesion	4
	Chromosome segregation	5	Post-embryonic organ morphogenesis	4
	Cellular component organization or biogenesis	5	Maintenance of location	3
	Regulation of chromosome segregation	5	Maintenance of protein location in extracellular region	3
	Negative regulation of blood coagulation	4		
	Response to hypoxia	4		

* *Gene ontology enrichment analysis was performed on differentially expressed genes in irradiated vs. sham-irradiated ECs (n = 3). Table shows the top 10 GO enrichment terms among upregulated (left) and downregulated (right) differentially expressed genes at day 1 (top) and at day 7 after 2 Gy of X-rays (bottom)*.

**Table 2:** Fe ion irradiation of ECs induces a gene expression signature suggesting persistent cell cycle block and decreased cellular adhesion.^*^

	Upregulated description of process	−log10 (*p*-value)	Downregulated description of process	−log10 (*p*-value)
2 Gy Fe ions Day 1	Signal transduction by p53 class mediator	11	Cell cycle process	99
	DNA damage response, signal transduction by p53 class mediator	7	Chromosome organization	67
	Positive regulation of cell cycle arrest	7	Cell division	49
	Response to radiation	7	DNA metabolic process	49
	Signal transduction in response to DNA damage	7	DNA replication	43
	Response to abiotic stimulus	7	Cellular component organization or biogenesis	38
	Regulation of cell proliferation	7	Chromosome segregation	26
	Regulation of apoptotic process	6	Microtubule-based process	19
	Response to stimulus	6	Regulation of chromosome segregation	18
	Chondroblast differentiation	5	Negative regulation of gene expression, epigenetic	17
2 Gy Fe ions Day 7	Developmental process	8	Regulation of endothelial cell migration	10
	Negative regulation of response to stimulus	8	Negative regulation of nucleic acid-templated transcription	10
	Regulation of locomotion	7	Regulation of signal transduction	9
	Regulation of cell proliferation	7	Developmental process	8
	Locomotion	7	Cytoskeleton organization	7
	Single-organism process	7	Response to endogenous stimulus	6
	Positive regulation of biological process	7	Regulation of cell death	6
	Single-organism developmental process	7	Regulation of cell cycle	6
	Regulation of multicellular organismal process	7	Regulation of cell proliferation	6
	Regulation of protein metabolic process	7	Cell adhesion	6

* *Gene ontology enrichment analysis (process) was performed on differentially expressed genes in irradiated vs. sham-irradiated ECs (n = 3). Table shows the top 10 GO enrichment terms among upregulated (left) and downregulated (right) differentially expressed genes on day 1 (top) or day 7 after 2 Gy of Fe ions (bottom)*.

### Fe ion-irradiated ECs have more differentially expressed proteins in comparison to X-rays, indicating a pronounced and persistent radiation response in comparison

**Figure 5:**
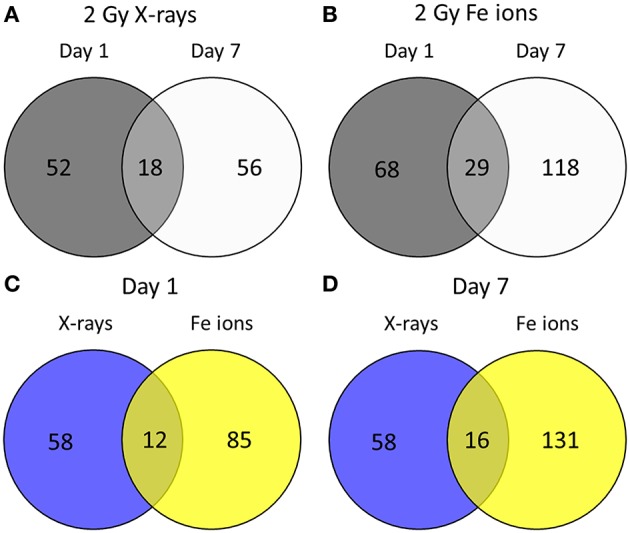
Irradiated ECs demonstrate a larger number of differentially expressed proteins after Fe ion exposure in comparison to X-ray exposure. Venn diagrams indicate the numbers of differentially expressed proteins after exposure to a single 2.0 Gy dose of X-rays **(A)** or Fe ions **(B)**. The protein expression changes after low and high LET irradiation are compared on day 1 **(C)** and on day 7 **(D)**. Changes are shown compared to protein expression in sham irradiated cells, as described in section Materials and Methods (*N* = 3, *n* = 12).

To determine if the proteomic effects in ECs are dependent on radiation quality, proteins derived from ECs irradiated with either a single 2 Gy Fe ion dose or a single 2 Gy X-ray dose were studied at 1 and 7 days after radiation exposure using a global proteomics analysis. The list of differentially expressed proteins is displayed in Supplementary Data File 3. The numbers of deregulated proteins using the two radiation qualities and time points are shown in Figure 5. While there was virtually no change in the number of differentially expressed proteins (up and down-regulated) between day 1 (70) and day 7 (74) after exposure to X-rays (Figure 5A), the number of differentially expressed proteins induced by Fe ion exposure almost doubled between day 1 and 7 (Figure 5B). In addition, compared to X-rays, Fe ions induced more differentially expressed proteins at day 1 (Figure 5C) and at day 7 post-irradiation (Figure 5D). The number of shared proteins between the two types of exposure was small, indicating a different protein fingerprint with the two radiation qualities in question.

### Although with different kinetics, Fe ions and X-ray irradiation both induce protein expression involved in caveolar mediated endocytosis signaling and cell-cell adhesion

**Figure 6:**
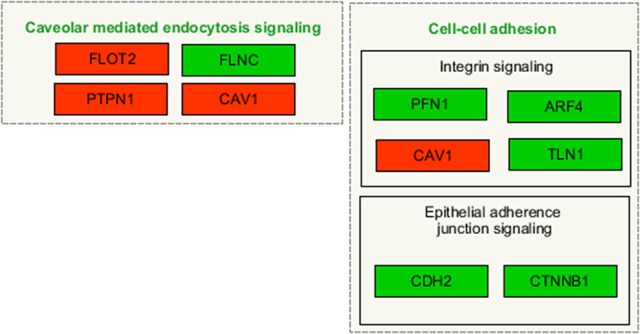
Irradiated ECs differentially expressed proteins after X-ray exposure involved in caveolar-mediated endocytosis and cell-cell adhesion. Arborescence diagram indicates caveolar-mediated endocytosis signaling and cell-cell adhesion pathways with the identified upregulated genes indicated in green and downregulated genes in red. Pathway diagram was adapted from Wikipathways and modified with Pathvisio. Changes are shown compared to gene expression in sham irradiated cells (*N* = 3, *n* = 12).

**Figure 7:**
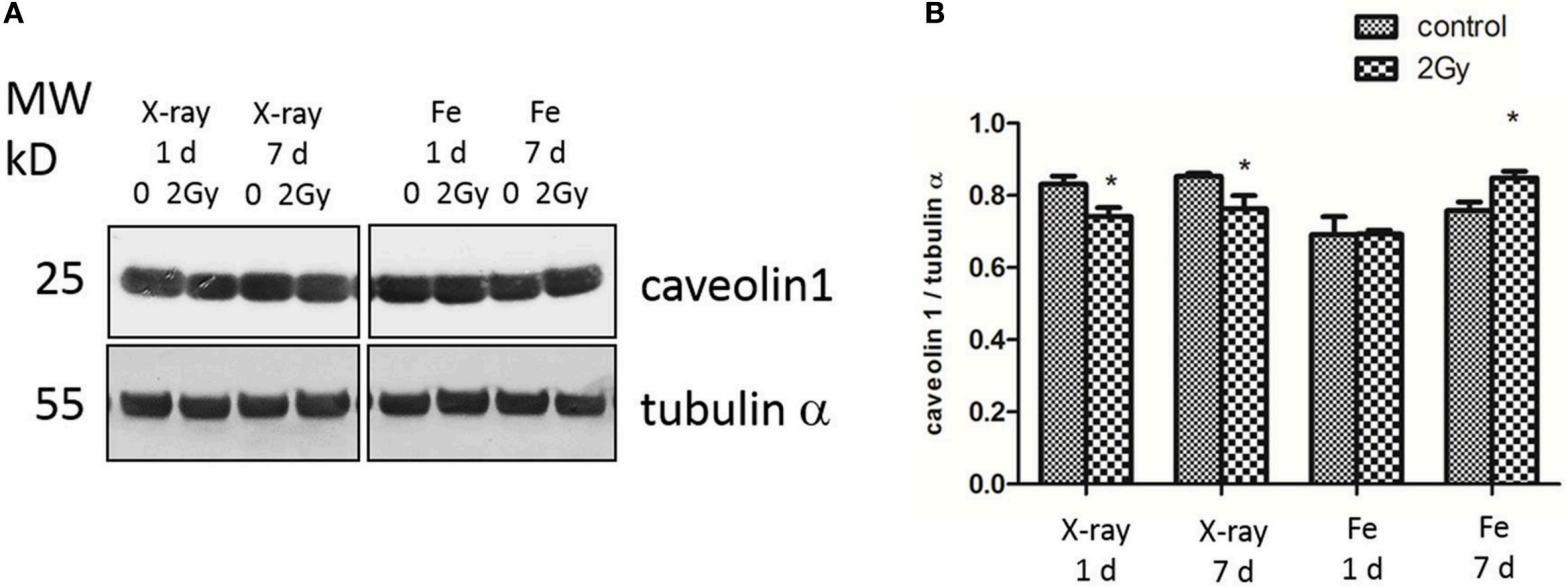
X-ray irradiated ECs exhibit a persistent downregulation of caveolin-1, whereas Fe ion exposure induces a caveolin 1 upregulation. **(A)** Caveolin-1 (cav-1) and α-tubulin protein expression analyzed using western blot after cell exposure to a single 2.0 Gy dose fo X-ray or Fe ions. **(B)** Data represent the cav-1/α-tubulin ratio in control and irradiated samples after background correction and normalization to α-tubulin expression. Data show means ± SEM (*N* = 3, *n* = 12). ^*^p < 0.05 using two-sided *t*-test (Welch-test).

At 1 day after exposure to a single X-ray dose of 2 Gy, a detailed analysis of altered biological pathways revealed that differentially expressed proteins played a role in caveolar mediated endocytosis signaling (FLOT2, FLNC, PTPN1, and CAV1; Figure 6) and in cell-cell adhesion, including integrin signaling (PFN1, ARF4, CAV1 and TLN1; Figure 6) and epithelial adherence junction signaling (CDH2 and CTNNB1; Figure 6) (Table 3, left panels and Supplementary Data File 4). Fe ion irradiation induced in general similar pathway changes, with differentially expressed proteins involved in endocytosis signaling and cell-cell adhesion, but at a later time point (day 7) (Table 3, right panels). While X-rays markedly deregulated proteins involved in endocytosis signaling at both days, Fe ions only induced a marked effect at day 7 and merely a small but significant effect on day 1. Furthermore, differentially expressed proteins involved in cell-cell adhesion were mostly observed at day 7 after X-ray exposure, whereas they were mostly observed on day 1 following Fe ion exposure (Table 3, right panels). In addition to analysis of altered pathways, differentially expressed proteins were classified based on their molecular function (Table 4 and Supplementary Data File 5). Proteomic alterations covered a broad range of cellular events, including cell death and survival, cell cycle, cellular movement and cell-cell signaling. In light of the bidirectional regulation of proteins, all the shown pathways were derived from the entire proteomics data as no separation was performed between up- and downregulated proteins. To better document alteration of caveolar- mediated endocytosis signaling, we focused on caveolin-1, which is the main protein component of caveolae (Frank, 2010). EC exposure to X-rays induced a persistent downregulation of caveolin 1 expression detected suing western blotting day 1 and day 7 (Figures 7A,B). Comparatively, Fe ions only upregulated of caveolin 1 expression at day 7 after exposure, indicating a difference between both radiation types.

**Table 3:** Fe ions and X-ray irradiation induces protein expression involved in caveolar mediated endocytosis signaling and cell-cell adhesion, but at different time points.^*^

	X-rays description of process	−log10 (*p*-value)	Fe ions description of process	−log10 (*p*-value)
Day 1	Caveolar-mediated Endocytosis Signaling	4.1	Remodeling of Epithelial Adherens Junctions	4.8
	RAN Signaling	2.9	tRNA Charging	4.5
	Glutathione Redox Reactions I	2.6	Glutathione-mediated Detoxification	3.5
	ILK Signaling	2.4	Integrin Signaling	3.3
	Virus Entry via Endocytic Pathways	2.4	Epithelial Adherens Junction Signaling	3.2
	Integrin Signaling	2.3	Mitochondrial Dysfunction	2.9
	Palmitate Biosynthesis I (Animals)	2.2	Germ Cell-Sertoli Cell Junction Signaling	2.9
	Uridine-5′-phosphate Biosynthesis	2.2	Oxidative Phosphorylation	2.8
	Fatty Acid Biosynthesis Initiation II	2.2	Paxillin Signaling	2.7
	Cell Cycle Control of Chromosomal Replication	2.2	EIF2 Signaling	2.7
Day 7	Remodeling of Epithelial Adherens Junctions	6.9	Caveolar-mediated Endocytosis Signaling	8.9
	Cell Cycle Control of Chromosomal Replication	5.1	Virus Entry via Endocytic Pathways	8.7
	Integrin Signaling	5.0	Neuroprotective Role of THOP1 in Alzheimer's Disease	6.5
	Epithelial Adherens Junction Signaling	5.0	Ephrin Receptor Signaling	5.5
	Gap Junction Signaling	4.6	Antigen Presentation Pathway	5.3
	Germ Cell-Sertoli Cell Junction Signaling	4.6	Phagosome maturation	5.2
	Sertoli Cell-Sertoli Cell Junction Signaling	4.5	NRF2-mediated Oxidative Stress Response	5.1
	Caveolar-mediated Endocytosis Signaling	4.0	Agrin Interactions at Neuromuscular Junction	5.1
	Death Receptor Signaling	3.6	Granzyme A Signaling	5.0
	Virus Entry via Endocytic Pathways	3.4	ERK/MAPK Signaling	5.0

* *Canonical pathway analysis was performed on differentially expressed proteins in irradiated vs. sham-irradiated ECs (n = 3). Table shows the top 10 molecular functions identified on day 1 (top) or day 7 (bottom) in ECs irradiated with 2 Gy of X-rays (left) or Fe ions (right)*.

**Table 4:** Differently expressed proteins after Fe ion and X-ray irradiation are mainly involved in cell death, cell cycle and cell-cell signaling.^*^

	X-rays description of molecular function	−log10 (*p*-value)	Fe ions description of molecular function	−log10 (*p*-value)
Day 1	Cell Death and Survival	6.8	Cell Death and Survival	10.3
	Cell Morphology	6.8	Cellular Growth and Proliferation	5.5
	Cell-To-Cell Signaling and Interaction	6.7	Cell Morphology	4.4
	Cellular Movement	6.5	Cellular Development	4.4
	Cellular Growth and Proliferation	6.1	Cellular Function and Maintenance	4.4
	Cellular Assembly and Organization	5.7	Cellular Assembly and Organization	4.4
	Cellular Compromise	5.7	Cell-To-Cell Signaling and Interaction	4.3
	DNA Replication. Recombination. and Repair	4.8	Cell Cycle	3.9
	Cellular Development	4.7	Cellular Movement	3.9
	Cell Cycle	4.1	Post-Translational Modification	3.8
Day 7	Cell Death and Survival	8.5	Cell Death and Survival	14.9
	Cellular Movement	6.4	Cell-To-Cell Signaling and Interaction	9.2
	Cell Morphology	6.2	Cellular Movement	7.6
	Cellular Growth and Proliferation	5.8	Cellular Growth and Proliferation	7.0
	Cell Cycle	5.4	Cell Cycle	6.5
	Cellular Function and Maintenance	5.0	Cellular Development	6.3
	Cell-To-Cell Signaling and Interaction	4.9	Cellular Function and Maintenance	5.7
	DNA Replication. Recombination. and Repair	4.8	Cell Morphology	5.3
	Cellular Development	4.3	Cellular Assembly and Organization	5.2
	Gene Expression	3.9	Protein Degradation	4.5

* *Protein ontology analysis was performed on differentially expressed proteins in irradiated vs. sham-irradiated ECs (n = 3). Table shows the top 10 molecular functions identified on day 1 (top) or day 7 (bottom) in ECs after a 2 Gy irradiation with X-ray (left) or Fe ions (right)*.

### X-ray and Fe ion exposure induces cytokine release and alters monocyte adhesion

**Figure 8:**
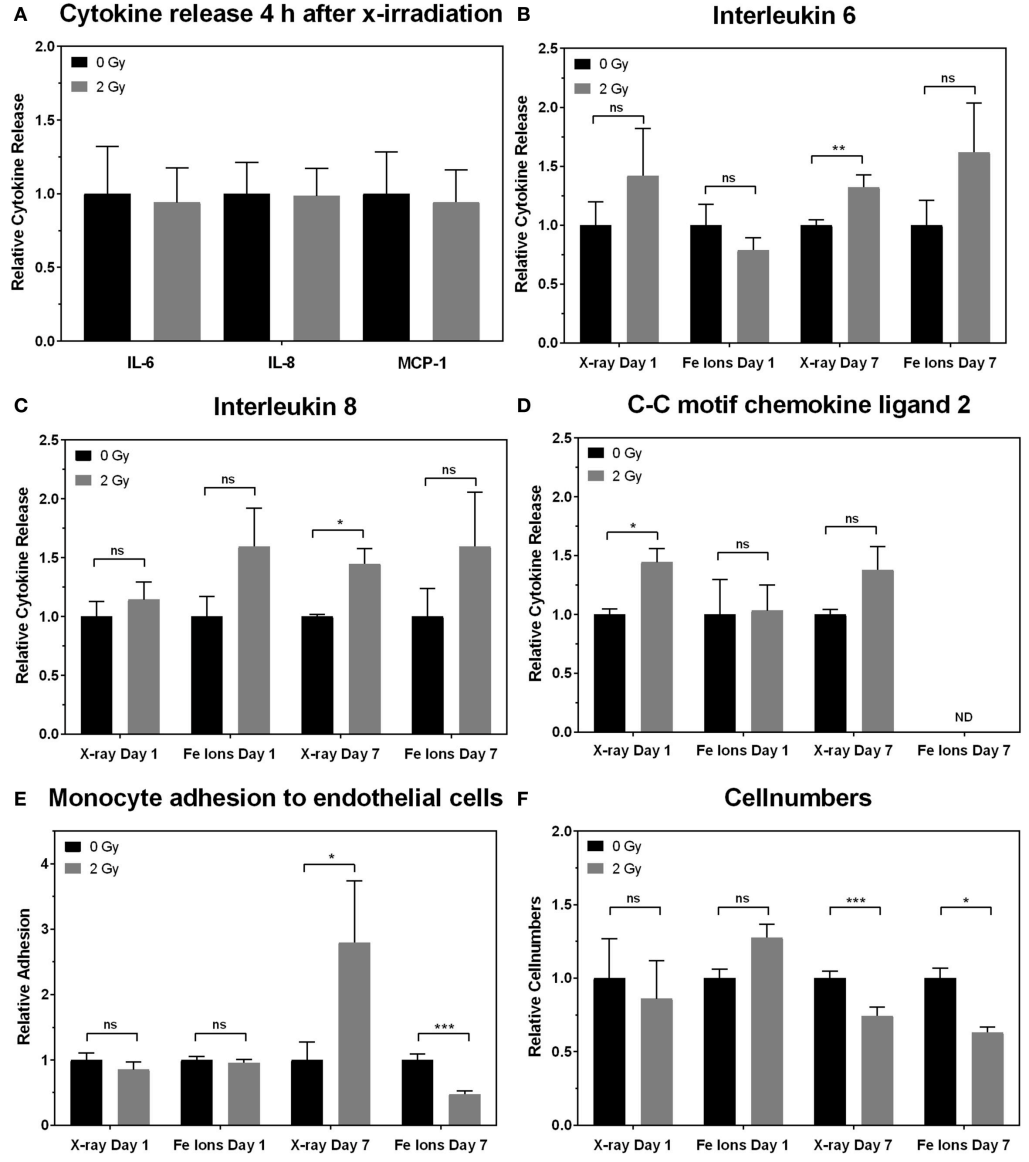
X-ray irradiation causes EC inflammation and adhesiveness to monocytes, whilst Fe ion irradiation reduces cellular number and decreases EC adhesiveness to monocytes. The levels of IL-6, IL-8, and CCL2 secreted by EC on 4 h and day 1 plus day 7 **(B–D)** are shown after exposure to X-rays (*n* = 18–27) and Fe ions (*n* = 4–6). Data were normalized to cell numbers, supernatant volume, and control values. **(E)** The numbers of monocytes adhering to EC on day 7 are shown after exposure to X-rays (*n* = 90–225) and Fe ions (*n* = 30). Data was normalized to cell numbers and control values of sham-irradiated ECs. **(F)** EC numbers on day 7 using either X-rays (*n* = 18–27) or Fe ions (*n* = 4–6) are shown. **(A–F)**. Data show mean ± SEM. ns, not significant, ^*^ *p* < 0.05, ^**^ *p* < 0.005, ^***^ *p* < 0.001 using two-sided *t*-test (Welch-test).

In order to study the functional effect of irradiation on inflammatory processes in ECs, we focused on 3 pro-inflammatory cytokines known to be involved in atherosclerosis: IL-6 (Schuett et al., 2009), IL-8 (Boisvert et al., 1998), and CCL2 (Harrington, 2000). At 4 h after X-ray exposure and on day 1 after exposure to either X-rays or Fe ions, there was no significant change in IL-6 and IL-8 levels measured using ELISA (Figures 8A–C). However, secreted CCL2 levels on day 1 increased only after exposure to X-rays. Seven days after exposure to a single dose of X-rays, ECs released more IL-6 (Figure 8B) and IL-8 (Figure 8C) but not CCL2 (Figure 8D) in comparison to control cells. In contrast, Fe ions did not significantly alter the secretion of IL-6 (Figure 8B) or IL-8 (Figure 8C), and CCL2 levels were below the detection threshold of ELISA assays (Figure 8D). When testing monocyte adhesion on ECs as a functional outcome of EC inflammation, X-rays were found to induce whilst Fe ions reduced monocyte adhesion on irradiated ECs 7 days post-irradiation (Figure 8E). Furthermore, irradiation with both X-rays and Fe ions significantly reduced the number of ECs 7 days after exposure (Figure 8F).

## Discussion

Our study aimed at investigating differences in signaling pathways and radiation response mechanisms in ECs irradiated with a single 2 Gy dose of either X-rays or Fe ions. We report time- and radiation type-dependent changes in the expression of genes and protein involved in cell cycle progression, cell-cell adhesion and caveolar-mediated endocytosis signaling. Furthermore, we found that X-ray irradiation induces EC release of pro-inflammatory cytokines IL-6 and IL-8 and increases monocyte adhesiveness of ECs, whilst Fe ions were found not to significantly induce cytokine secretion and reduced monocyte adhesiveness of ECs. These changes are indicative of a radiation quality-dependent changes in ECs, which can be linked to atherosclerosis and underscores the importance of conducting future research to better understand processes altered in healthy tissues exposed to high LET radiation.

It is generally accepted that high LET radiation (e.g., Fe ions) has a higher RBE in comparison to low LET radiation (e.g., X-rays) (ICRP, 2007). In accordance, we found that the transcriptional and proteomic impact of irradiation was more pronounced and longer lasting for Fe ions in comparison to X-rays. At the gene expression level, both radiation qualities were shown to repress the expression of genes involved in cell cycling on day 1, as previously demonstrated for X-rays using the same *in vitro* model (Baselet et al., 2017). However, X-ray-irradiated ECs induced the expression of cell-cycle genes on day 7, indicating a reactivation of the cell cycle process (Baselet et al., 2017). In contrast, Fe ion irradiation persistently repressed the expression of genes associated with cell cycle regulation. A permanent cell cycle arrest after high LET exposure has also been demonstrated by others (Fournier and Taucher-Scholz, 2004; Suetens et al., 2016) and is believed to be a consequence of the complex, clustered hard-to-repair DNA damage it induces (Hada and Georgakilas, 2008; Asaithamby and Chen, 2011).

The observed changes of transcriptional and proteomic signaling involved in adhesion pathways in irradiated ECs may have two biological interpretations. Diminished EC adhesion signaling, as observed on day 7 after irradiation with both X-rays and Fe ions, can be a result of angiogenic activation, a process initiating vascular extension from preexisting blood vessels and maintained by proliferating ECs (Folkman, 1971). Emerging evidence indicates that high LET inhibits (Takahashi et al., 2003; Mao et al., 2010; Grabham et al., 2013) and low LET radiation promotes angiogenesis (Sonveaux et al., 2003; Sofia Vala et al., 2010). However, angiogenic activation is less likely to have occurred after X-ray and Fe ion irradiation in our model because cell numbers were decreased. Cell number reduction in Fe ion-treated samples was accompanied by decreased transcriptional and proteomic signaling linked to EC death and reduced proliferation, making angiogenic activation even less likely. A more likely explanation would be compromised EC integrity, corroborated by the observed decrease in EC number and adhesiveness in combination with signaling linked to increased EC death and reduced proliferation. In line with this, nickel ion radiation of ECs *in vitro* was found to induce the expression of genes involved in endothelial permeability and apoptosis (Beck et al., 2014). Furthermore, Fe ions induced a decreased EC number *in vivo* 12 months after exposure to a single 2 Gy dose (Mao et al., 2010). Loss of EC integrity may induce a number of adverse effects, including thrombus formation, and could predispose to the development of chronic pathologies, such as atherosclerosis leading to CVD (Widlansky et al., 2003; Deanfield et al., 2007; Munzel et al., 2008).

Another observation is the potential involvement of caveolar mediated endocytosis signaling in the EC response to IR exposure. Indeed, caveolin-1 (cav-1) protein expression was decreased on days 1 and 7 after X-ray exposure, whereas Fe ion exposure increased cav-1 expression on day 7. During endocytosis, plasma membrane invagination leads to the formation of intracellular carrier vesicles that allow the engulfment of particles or fluids from the extracellular environment (de Duve, 1983). Although this process is known to occur during oxidative stress in ECs (Sundqvist and Liu, 1993; Liu and Sundqvist, 1995), less is known about caveolar mediated endocytosis during cellular responses to IR. The principal component protein of caveolae, caveolin-1 (Cav-1), has been shown to play a role in DNA damage repair (Zhu et al., 2010) and is therefore related to EC radiosensitivity (Klein et al., 2015). In addition, Caveolin 1 expression has been shown to suppress cell-cycle progression (Hulit et al., 2000) and inhibits angiogenesis (Liu et al., 1999; Morais et al., 2012) by mediating contact inhibition (Galbiati et al., 1998) and by reducing endothelial nitric oxide synthase abundance and activity (Sbaa et al., 2005). Consequently, an elevated endothelial expression of Cav-1 has been linked to processes underlying the development and progression of atherosclerosis (Fernandez-Hernando et al., 2010; Pavlides et al., 2014).

Inflammation plays a central role in the development, progression and final outcome of atherosclerosis (Libby, 2002). In this study, we evidenced that a single 2 Gy X-ray dose induces inflammation in ECs at day 7 after exposure, whereas 2 Gy Fe ions did not significantly induce inflammation. The pro-inflammatory effect of X-ray exposure was previously evidenced in the same *in vitro* model (Baselet et al., 2017). In accordance, others observed elevated IL-6 and IL-8 levels in 2–10 Gy X-ray-irradiated EC cultures (Meeren et al., 1997), elevated IL-6 levels in EC cultures after chronic irradiation with a total dose of 2 Gy (Ebrahimian et al., 2015) and elevated blood levels of IL-6 were detected in A-bomb survivors (Hayashi et al., 2003). These changes were previously demonstrated to increase EC adhesiveness to monocytes 1 day after exposure to X-ray doses higher than 5 Gy (Khaled et al., 2012). In contrast, reduced EC adhesiveness to monocytes in response to high LET IR is more controversial, since others have reported increased monocyte adherence to ECs 1 day after 2 and 5 Gy Fe ion exposure (Kucik et al., 2010; Khaled, 2011). In these studies, ECs were pretreated with tumor necrosis factor α, known to induce EC adhesiveness to monocytes (Ikuta et al., 1991; Mackay et al., 1993), which may have caused a bias. Nonetheless, these authors used a flow chamber system that provides an environment with fluid shear stress resembling the *in vivo* blood vessel environment. In contrast to our findings, high LET radiation exposure has been shown to increase the *in vitro* expression of adhesion molecules (Kiyohara et al., 2011), which can lead to accelerated development and progression of atherosclerosis in obese apoE^−/−^mice (Yu et al., 2011). These conflicting results highlight the need for further research with a particular focus on radiation dose and timing after exposure to univocally determine the effects of high LET radiation on EC inflammation in the frame of CVD.

In this study, we determined the effects of radiation quality on human coronary artery ECs seeing their importance in radiation-induced CVD (Vita and Keaney, 2002; Darby et al., 2013; Widmer and Lerman, 2014). As the Fe ions in this study were accelerated to 1 GeV and had a penetration depth of up to 25 cm in water (Scholz, 2003; Lee et al., 2011), they are able to reach endothelial linings in the human body since tissue is generally considered to be similar to water. Although immortalized, these cells retain a normal EC phenotype, including genome stability (unpublished data), normal cell cycle regulation (Baselet et al., 2017), responsiveness to IR (Lowe and Raj, 2014) and the expression of all major EC phenotypic markers (e.g., von Willebrand factor and cadherin-5; unpublished data). Due to the large phenotypic heterogeneity between ECs derived from different vascular beds, care should be taken before generalizing our findings (Aird, 2007a,b). Furthermore, our *in vitro* model is not adapted to provide the entire complexity of the development of radiation-induced CVD in humans. Although Fe ions are a form of high LET radiation that is more relevant for the space environment than medical practice, they can provide valuable insights into the endothelial response to high LET exposure.

In conclusion, we found a time- and radiation quality dependent endothelial radiation response. In general, the radiation impact was more pronounced and longer lasting for Fe ions than for X-rays. Observed transcriptional and proteomic changes were involved in cell cycle regulation, cell death, caveolar mediated endocytosis signaling and EC permeability. In contrast to Fe ions, X-rays were found to induce EC inflammation and adhesiveness to monocytes. Besides the link with CVD development and progression, these findings are indicative of a different molecular response induced by the two types of irradiation. In the context of the ProCardio FP7 project, our findings will be integrated with other *in vitro, in vivo* and epidemiological data to increase our understanding of radiation-induced CVD. Although studies on the effect of high LET particles are scarce due to limited access to the radiation facilities, future research should continue aiming at elucidating the underlying molecular mechanisms induced by high LET radiation in ECs. Before exploring details of each modified pathway, our findings should be confirmed with independent irradiated EC samples. Besides the quality effect, emphasis should also be placed on the dose effect, as low and high dose irradiation may have different outcomes. Furthermore, radiation doses of Fe ions and X-rays should be identified with an equal RBE in order to compare cellular responses to the 2 radiation types on the same scale of biological damage. A better understanding of the radiation-induced CVD risk is necessary for the protection of radiotherapy patients but also astronauts in space.

## Author contributions

BB performed microarray analysis (functional enrichment analysis). Microarrays were performed by AJ and AM. OA and MVB performed the proteomic analysis. NE, TD, and SK quantified cytokines and endothelial adhesiveness. DL and KR created and validated the human coronary artery EC line. All authors helped with data interpretation, scientific guidance and preparation of the manuscript.

### Conflict of interest statement

The authors declare that the research was conducted in the absence of any commercial or financial relationships that could be construed as a potential conflict of interest.
